# Effects of Dietary Lysophospholipid Inclusion on the Growth Performance, Nutrient Digestibility, Nitrogen Utilization, and Blood Metabolites of Finishing Beef Cattle

**DOI:** 10.3390/antiox11081486

**Published:** 2022-07-29

**Authors:** Meimei Zhang, Haixin Bai, Yufan Zhao, Ruixue Wang, Guanglei Li, Guangning Zhang, Yonggen Zhang

**Affiliations:** College of Animal Science and Technology, Northeast Agricultural University, Harbin 150030, China; mmzhang12@126.com (M.Z.); baihaixin666@163.com (H.B.); zyf19994268@163.com (Y.Z.); wrx983942@163.com (R.W.); liguanglei20220729@163.com (G.L.)

**Keywords:** Angus beef bulls, blood metabolites, digestibility, growth performance, lysophospholipid

## Abstract

This study was conducted to evaluate the effect of dietary supplementation with lysophospholipids (LPLs) on the growth performance, nutrient digestibility, nitrogen utilization, and blood metabolites of finishing beef cattle. In total, 40 Angus beef bulls were blocked for body weight (447 ± 9.64 kg) and age (420 ± 6.1 days) and randomly assigned to one of four treatments (10 beef cattle per treatment): (1) control (CON; basal diet); (2) LLPL (CON supplemented with 0.012% dietary LPL, dry matter (DM) basis); (3) MLPL (CON supplemented with 0.024% dietary LPL, DM basis); and (4) HLPL (CON supplemented with 0.048% dietary LPLs, DM basis). The results showed that dietary supplementation with LPLs linearly increased the average daily gain (*p* < 0.01), digestibility of DM (*p* < 0.01), crude protein (*p* < 0.01), and ether extract (*p* < 0.01) and decreased the feed conversion ratio (*p* < 0.01). A linear increase in N retention (*p =* 0.01) and a decrease in urinary (*p* = 0.04) and fecal N (*p* = 0.02) levels were observed with increasing the supplemental doses of LPLs. Bulls fed LPLs showed a linear increase in glutathione peroxidase (*p* = 0.04) and hepatic lipase (*p* < 0.01) activity and a decrease in cholesterol (*p* < 0.01), triglyceride (*p* < 0.01), and malondialdehyde (*p* < 0.01) levels. In conclusion, supplementation with LPLs has the potential to improve the growth performance, nutrient digestibility, and antioxidant status of beef cattle.

## 1. Introduction

Lipids (fats and oils) are commonly added to livestock diets as a concentrated energy source and have the highest caloric value of all nutrients [1]. Fats provide approximately 2.25-fold more energy available to animals than carbohydrates and proteins. The dietary fat supplementation of ruminants has been studied as a means to affect various physiological processes or change the fatty acid composition of ruminant feed [2]. Fats also provide essential fatty acids, which are carriers of fat-soluble vitamins and play important roles in biochemistry, physiology, and nutrition [3,4]. Lipids are water-insoluble substances that are decomposed into small molecules through bile-mediated emulsification, decomposed into glycerol and fatty acids under the action of lipase, and digested and absorbed via the gastrointestinal tract [5]. However, diets with a high proportion of lipids require far greater levels of bile acid secretion by animals; the incomplete emulsification of lipids and insufficient lipase secretion in the body not only causes resource waste but also endangers the health of livestock and poultry. Studies have reported that the limitation of lipase activity may prevent the formation of mixed micelles in the intestinal lumen, thereby further reducing fat digestion and the absorption of nutrients [6,7]. Furthermore, although supplementation with fat to fatten cattle diets could increase live weight gain, incomplete fat absorption would lead to an increase in feeding cost [8]. Therefore, it is necessary to add exogenous emulsifiers to the diet to promote the digestion and absorption of lipids.

Lysophospholipids, as promising feed additives, have been widely used in nonruminant animals to improve growth performance, feed efficiency, and dietary fat absorption when diets supplemented with LPLs are fed to pigs and poultry [1,9]. Brautigan et al. [9] found that LPLs increased dietary fat absorption due to their emulsification property and upregulation of the expression of various genes, such as GAS6 and RAMP2, in the intestinal epithelium. However, to the best of our knowledge, research on the effects of LPL inclusion in ruminants is still limited. As feed additives for dairy cows and lambs, LPLs improved the gain-to-feed ratio and growth performance [10,11]. Reis et al. [12] found that the inclusion of LPLs as feed additives in milk replacers at a dose of 4 g/d increased the average daily gain (ADG), feed efficiency, and fecal score of dairy calves. Supplementation of LPLs in the diet linearly increased the milk yield, feed efficiency (milk yield/DM intake), and milk protein and fat yields of dairy cows [13]. In addition, studies have reported that phospholipids (source of LPLs) in the rumen can escape microbial degradation and increase emulsification in the small intestine [13,14]. However, the literature on the evaluation of LPLs to improve beef cattle production and feed efficiency is scarce. We hypothesized that LPLs would improve growth production and feed efficiency. Therefore, the objective of the current study was to investigate the effects of LPLs as feed additives on growth performance, nutrient digestibility, N utilization, and blood metabolites in beef cattle.

## 2. Materials and Methods

The study received approval from the Institutional Animal Care Committee, Northeast Agricultural University (Harbin, China), and all experimental procedures were performed in accordance with the university’s guidelines for animal research.

### 2.1. Lysophospholipid Products

The LPL product used in the current experiment was hydrolyzed soy lecithin, including phospholipids, free fatty acids, and LPLs (16%), and was provided by the Guangdong Baimeiyide Biological Technology Co., Ltd. (Guangzhou, China). The main components of LPLs are 1-lysophosphatidylcholine (1-LPC), 2-lysophosphatidylcholine (2-LPC), lysophosphatidylethanolamine (LPE), lysophosphatidylinositol (LPI), and lysophosphatidic acid (LPA).

### 2.2. Animals, Experimental Design, and Diets

A total of 40 Angus beef bulls were blocked into 10 groups based on body weight (BW) (447 ± 9.64 kg) and age (420 ± 6.1 days), and bulls within a block were randomly allocated to 1 of 4 treatments. The treatments were as follows: (1) control (CON; basal diet); (2) LLPL (CON supplemented with 0.012% dietary LPL, dry matter (DM) basis); (3) MLPL (CON supplemented with 0.024% dietary LPL, DM basis); and (4) HLPL (CON supplemented with 0.048% dietary, DM basis). All beef cattle were housed individually in pens (4 × 3 m^2^) with free access to water and fed ad libitum. The entire experiment lasted for 9 weeks, including 2 weeks for adaptation and 7 weeks for data and sample collection. During the adaptation period, the feed intake of concentrate was gradually increased until the ratio of concentrate to roughage in the final diet (the first 10 days) reached 75:25, and then the feed intake was gradually increased until it reached an arbitrary feed intake. All the beef cattle were fed twice a day at 06:00 h and 18:00 h. The dietary ingredients and chemical composition are presented in Table 1.

### 2.3. Sample Collection

The feed offered to each beef cattle was recorded daily, and refusals were measured weekly during the sample collection period to calculate the dry matter intake (DMI). The body weights of the beef cattle were measured at the beginning of the collection period on 2 consecutive days (week 0), at the midpoint on 2 consecutive days (week 4), and off test on 2 consecutive days (week 7) before the morning feeding to determine the average daily gain (ADG). Samples of individual feed ingredients, refusals, and diets were collected weekly for DM determination and were preserved for further analyses.

Fecal samples (approximately 500 g) were collected from the rectum during the final three days of weeks 4 and 7 daily at 0600, 1200, 1800, and 2400 h, and the samples were pooled by beef cattle and sampling day. The collected fecal samples were immediately dried at 55 °C for 48 h, ground through a 1 mm screen, and stored at 4 °C for subsequent chemical analysis.

Complete urine samples were collected during the final five days of weeks 4 and 7. The urine from each steer was collected using a funnel collector. One end of the funnel collector piped the urine directly into the container through a polyethylene tube, and the other end was fixed at the urination place of the steers as described by Alves et al. [16]. The container contained 500 mL of H_2_SO_4_ (200 mL/L) to prevent nitrogen loss. The total urine volume was measured after a 24 h period, and the urine was stored at −20 °C until subsequent analysis.

Blood samples of all the beef cattle were collected in sodium heparin tubes from the tail vein before morning feeding on the last day of weeks 4 and 7. Subsequently, blood samples were centrifuged (3000× *g*, 20 min, 4 °C) to obtain supernatants of plasma and then stored at −20 °C until analysis.

### 2.4. Chemical Analyses

All feed samples, refusals, and thawed fecal samples were dried in an air-forced oven at 55 °C for 48 h for DM (method 934.01), ether extract (EE; 920.39), and ash (method 942.05) determination following the methods of AOAC International (2000) [17]. Crude protein was analyzed from the nitrogen content multiplied by 6.25 according to AOAC (2000) [17]. The neutral detergent fiber (NDF) and acid detergent fiber (ADF) contents were determined using an Ankom 220 fiber analyzer (Ankom Technology, Macedon, NY, USA) following the methods of Van Soest et al. [18]. NDF was analyzed using heat-stable α-amylase and sodium sulfite. The apparent total tract digestibility of nutrients was determined by using the acid detergent-insoluble ash content of feeds and feces as an internal marker, as described by Van Keulen and Young [19], and the formula was described according to Zhong et al. [20].

The concentrations of total protein (TP), albumin (ALB), globulin (GLB), aspartate aminotransferase (AST), alanine aminotransferase (ALT), alkaline phosphatase (ALP), glucose (GLU), creatinine (CRE), total serum cholesterol (CHOL), triglyceride (TG), high-density lipoprotein cholesterol (HDL-C), low-density lipoprotein cholesterol (LDL-C), uric acid (UA), and urea nitrogen (BUN) were analyzed in Heilongjiang Electric Power Hospital (Harbin, China) by a fully automatic biochemical analyzer (Roche Cobus Mira Plus, Cham, Switzerland) using commercial diagnostic kits supplied by the Nanjing Jian Cheng Bioengineering Institute (Nanjing, China). Commercially available kits (Nanjing Jian Cheng Bioengineering Institute, Nanjing, China) were used to measure the concentrations of plasma total antioxidant capacity (T-AOC) and the malondialdehyde (MDA), total superoxide dismutase (T-SOD), glutathione peroxidase (GSH-Px), lipoprotein lipase, and hepatic lipase (HL) levels.

### 2.5. Statistical Analyses

All data were analyzed using the Proc Mixed procedure of SAS (version 9.2; SAS Institute Inc., Cary, NC, USA). The statistical model included week, treatment, and interaction of treatment × week as fixed effects; the week was treated as a repeat measurement, and beef cattle within treatment was treated as a random effect. Linear and quadratic orthogonal polynomial contrasts were used to analyze the effect of increasing the LPL dose. Significant differences were declared at *p* ≤ 0.05, and trends were defined at 0.05 < *p* ≤ 0.10.

## 3. Results

### 3.1. Growth Performance and Nutrient Digestibility

The growth performance and digestibility of beef cattle are presented in Table 2. The DMI and ME intakes were similar among the dietary treatment groups. The ADG (*p <* 0.01) linearly increased, and the feed conversion ratio (FCR) (*p <* 0.01) linearly decreased with increasing supplemental doses of LPLs. No significant difference was observed in the apparent digestibility of NDF and ADF with LPL supplementation. However, the digestibility of DM, EE, and CP linearly increased (*p <* 0.01) with increasing doses of LPLs. Moreover, the ADG (*p* < 0.01) and digestibility of DM (*p* < 0.01), EE (*p* < 0.01), and CP (*p* < 0.01) were higher, while the FCR (*p* < 0.01) was lower for LPLs than for CON.

### 3.2. Nitrogen Balance

The results of nitrogen utilization are presented in Table 3. Nitrogen intake was not affected by LPLs. Increasing the dose of LPLs in diets linearly decreased fecal N excretion (*p* = 0.02), that as a proportion of N intake (*p* < 0.01), and urinary N excretion (*p* = 0.04). Moreover, N retention (*p* = 0.01) and that as a proportion of N intake (*p* < 0.01) linearly increased with increasing doses of LPLs. Compared with CON, supplementation with LPLs resulted in higher fecal N excretion (*p* = 0.05) and lower N retention (*p* = 0.04).

### 3.3. Blood Metabolites

The results of plasma metabolite measurement are presented in Table 4. No significant differences in plasma GLB, AST, ALP, ALT, CRE, GLU, UA, or BUN concentrations were observed with increasing supplemental doses of LPLs. The concentrations of ALB quadratically (*p* < 0.01) increased with increasing supplemental doses of LPLs in diets. Moreover, linear effects were observed on the concentrations of TP (*p* = 0.01), TG (*p* < 0.01), CHOL (*p* < 0.01), LDL-C (*p* < 0.01), and HDL-C (*p* < 0.01) with LPL supplementation. The concentrations of TG (*p* = 0.02), CHOL (*p* = 0.04), and LDL-C (*p* < 0.01) were lower, whereas the concentrations of ALB (*p* < 0.01) and HDL-C (*p* < 0.01) were higher with LPL supplementation than in CON.

### 3.4. Antioxidant Function and Enzyme Activities

The results of the antioxidant function and enzyme activity analyses are presented in Table 5. A quadratic increase in the activities of T-SOD (*p* = 0.03) and lipoprotein lipase (*p* < 0.01) was observed with increasing the doses of LPLs. Moreover, the activities of GSH-Px (*p* = 0.04), TL (*p* < 0.01), and HL (*p* < 0.01) linearly increased with increasing the supplemental doses of LPLs. A quadratic decrease (*p* < 0.01) in the MDA level was observed with increasing the supplemental doses of LPLs. In comparison with CON, supplementation with LPLs increased the activities of TL (*p* < 0.01), lipoprotein lipase (*p* < 0.01), and HL (*p* < 0.01) and decreased the concentration of MDA (*p* < 0.01).

## 4. Discussion

### 4.1. Growth Performance

In the current study, the beef cattle fed diets supplemented with LPLs exhibited a higher ADG and lower FCR, indicating an improvement in growth performance. A recent experiment conducted by Reis et al. [12] showed that supplementation with LPLs improved growth performance and feed efficiency without affecting the DMI of dairy cows. Moreover, Chen et al. [21] found that steers fed diets that contained 1.0% lecithin exhibited higher ADG compared with the control group. In addition, LPL supplementation in lamb diets increased the ADG without affecting feed intake, which is consistent with the current findings [11]. The improved performance of beef cattle with LPL supplementation may have been due to the increase in the absorption of nutrients in the small intestine in the current study [22]. Gut microbial butyrate metabolic pathways have been reported to increase energy intake and improve intestinal histology (e.g., villi length and crypt depth) in livestock, thereby improving body weight [23,24]. Qiu et al. [25] reported that choline, as one of the main components of LPLs, increased the concentration of butyrate in the colon digesta of weaned piglets, which was positively correlated with the body weight of the animals. Therefore, we suspected that the mechanism for LPLs improving ADG might be due to the increased concentration of butyric acid in gut microorganisms, which has also been verified in our previous study [26]. In a study by Song et al. [27], however, Hanwoo heifers fed a diet with LPLs (0.3% or 0.5% *w*/*w*) did not exhibit altered growth performance. This discrepancy could be partially attributed to the different LPL products, sources of phospholipids, enzymatic (phospholipase) hydrolysis processes to produce LPLs, and proportions of LPLs in the product. In addition, the dosage level of LPLs was greater in the study by Song et al. [27] compared with our results. High doses of LPLs may have no effect on the growth performance of cattle. It is worth noting that LPL as an additive has widely been used in nonruminants, and the consistent effects of LPLs on animal production have been observed, which indicates that the degree of the ruminal bypass of LPLs might be critical for the effects of LPLs on the growth performance of beef cattle [1,14]. Jenkins et al. [14] reported that some phospholipids could escape rumen degradation and increase emulsification in the small intestine.

### 4.2. Nutrient Digestibility

Numerous studies on in vitro digestion have shown that dietary emulsifiers can modulate the direct contact of lipid substrates and lipase and thus promote lipid digestion [28,29]. In the current study, increasing the LPL in the diet linearly increased the digestibility of DM, EE, and CP. The beneficial effect of LPLs on EE digestibility may be explained by the emulsification property of LPLs. As a powerful surfactant, LPLs can effectively reduce the size of fat globules and promote the enzymatic hydrolysis of fat [12]. Previous studies reported that LPLs could modify the lipid bilayer of the membrane, altering the fluidity of the membrane and the transmembrane permeability of nutrients, thus promoting the digestibility of nutrients [30,31]. In addition, LPLs can change the formation of protein channels in the membranes of the lower gastrointestinal tract, increase the size and number of membrane pores, and thus improve the permeability of macromolecules across the cell membrane [32]. Consistent with our results, Song et al. [27] reported an improvement in the nutrient digestibility of heifers with the dietary inclusion of LPLs. Similarly, Huo et al. [33] demonstrated that the addition of LPLs to diets improved the nutrient digestibility of DM and CP in lambs. However, LPL supplementation in ruminants does not always respond uniformly to nutrient digestibility. A study from Lee et al. [13] found that dairy cows fed diets supplemented with LPLs tended to exhibit a decreased digestibility of DM and OM. The inconsistent results could be partially attributed to differences in diet, genetics, and enzymatic (phospholipase) hydrolysis processes to produce LPLs. In addition, studies on monogastric animals found that different sources of dietary lipids also affect the nutrient digestibility of LPLs [1]. For instance, when LPLs were added to the tallow, the digestibility increased; however, LPLs added to the lard decreased the digestibility [34].

### 4.3. Nitrogen Balance

Fecal nitrogen is mainly composed of undigested feed nitrogen and endogenous nitrogen [35]. Fecal nitrogen and urinary nitrogen levels are related to the digestion and absorption of amino acids by the small intestine [36]. In the current study, supplemental LPLs in diets decreased fecal N excretion and urinary N excretion, indicating that LPLs increased the N utilization rate. This is consistent with the study of Lee et al. [13], who also found that dairy cows fed diets supplemented with LPLs had more digestible amino acids or peptides in the gut, resulting in decreased urinary N excretion. Moreover, Brautigan et al. [9] found that adding LPLs to chicken diets upregulated various genes involved in nutrient absorption in intestinal epithelial cells and increased the length and width of intestinal villi, thereby increasing nutrient absorption. In the current study, the decreased N in feces and urine could be attributed to the promotion of amino acid absorption in the small intestine, and the result is also supported by the improved digestibility of CP.

### 4.4. Blood Metabolites

The concentrations of CHOL, HDL-C, LDL-C, and TG in plasma are important indicators of fat metabolism as well as fat and carbohydrate digestion. Our results showed that the inclusion of LPLs in the diet decreased the concentrations of CHOL and TG but increased the concentrations of TP and ALB. Consistently, He et al. [37] reported that adding 0.5 g/kg LPL to diets increased the concentration of TP and decreased the concentration of CHOL in dairy cows. In general, fats, which contain high SFAs, increase the levels of TG and CHO [38,39]. The supplementation of emulsifiers may lower the concentrations of TG and CHOL by using energy efficiently [40]. LPLs can clear chylomicrons from the blood faster or slow their release into the blood, thereby reducing the concentration of TG [41]. Jones et al. [34] suggested that pigs fed lecithin had decreased serum concentrations of TG, which may be due to the faster absorption and metabolism of the consumed fat. In this study, the decreased concentration of TG with LPL supplementation could be explained by the enhanced activity of lipoprotein lipase. Lipoprotein lipase can reduce plasma triglyceride levels and enhance lipid uptake and deposition by hydrolyzing TG on chylomicrons and very low-density lipoprotein [42]. Conversely, research from Li et al. [43] found that TG and CHO were increased when beef steers diets were supplemented with lecithin. The discrepancy between Li et al. [43] and the current study is difficult to explain. The mechanism by which LPLs influence TG or CHOL is still unclear. Additional studies are needed to determine the mechanism by which emulsifiers affect blood metabolites.

High-density lipoprotein cholesterol and LDL-C are the main transport proteins of cholesterol; the former is mainly responsible for transporting cholesterol from the blood into the liver, while the latter is mainly responsible for transporting cholesterol from the liver into the blood [44]. In the current study, supplementation with LPLs in diets decreased the concentration of LDL-C and increased the concentration of HDL-C, indicating that LPLs have regulatory effects on CHOL. Our results are consistent with those of a previous report by Jones et al. [34] that pigs fed lecithin or lysophosphatidic diets had lower LDL concentrations than those given treatments without emulsifiers. The mechanism for the HDL increase may be that LPLs decrease the lecithin–cholesterol acyltransferase activity in plasma, which could catalyze the transfer of the *sn*-2 acyl chain from phosphatidylcholine of HDL to the 3-hydroxyl group of unesterified cholesterol [45]. TP and ALB are synthesized mainly by the liver and are important indicators reflecting the absorption and metabolism status of proteins in the body as well as protein synthesis capacity in the liver [46]. In the current study, bulls fed LPLs showed a linear increase in TP and ALB concentrations, indicating that protein metabolism and liver function were affected by LPL supplementation. Reis et al. [12] reported that adding LPLs to milk replacer could increase the serum TP concentration of calves, possibly because LPLs changed the membrane structure and increased nutrient efficiency, thus increasing the TP concentration.

### 4.5. Antioxidant Function and Enzyme Activities

Oxidative stress caused by the imbalance of reactive oxygen species (ROS) can cause tissue damage and the loss of normal cell functions in cattle [47]. Some studies have shown that excess fat in the diet can lead to an imbalance in energy metabolism, the deposition of harmful lipids, and lipid peroxidation, eventually resulting in liver damage, inflammation, apoptosis, and ROS production [11,48]. The T-AOC, T-SOD, and GSH-PX in the endogenous antioxidant defense system can scavenge various ROS in the body and protect cells from oxidative damage [49]. Previous studies have shown that supplemental bile acid, which has similar functions to LPLs, could increase the activities of SOD, GSH-Px, and CAT and alleviate the damage to the antioxidant system caused by high fat levels [50]. Furthermore, Huang et al. [51] found in vitro that milk phospholipids can significantly improve antioxidant activity and delay the oxidation of PUFAs. In this study, supplemental LPLs increased the activities of SOD and GSH-Px in plasma, indicating that LPLs could promote antioxidant capacity and alleviate the damage caused to the antioxidant system of beef cattle. The effects of LPLs on antioxidative function may be related to their choline component, which reduces oxidative stress by modulating the redox status of the cell and inhibits the inflammatory response [52]. Choline supplementation alters the plasma methionine homocysteine circulating metabolite, resulting in an increase in S-adenosylmethionine (SAM) [53]. The elevation of SAM can prevent the induction of inducible nitric oxide synthase and increase the production of glutathione [54]. MDA, a product of oxygen radical-induced lipid peroxidation, is an important indicator for evaluating tissue oxidative stress damage [55]. Cai et al. [56] found that there is a negative correlation between dietary lecithin and MDA levels; as the phospholipid content increases, the MDA content decreases. Consistent with this, a negative correlation was observed between dietary LPLs and serum MDA levels in the current study. Lipoprotein lipase and HL are key enzymes that affect the delivery of fatty acids to tissues through hydrolysis of triglycerides [57]. Studies have demonstrated that lipoprotein lipase and HL are two important markers for measuring lipid metabolism ability and could also prevent the accumulation of excessive cholesterol in extrahepatic tissues [58]. In the present study, dietary supplementation with LPLs increased the activities of lipoprotein lipase, HL, and TL, indicating that LPLs can promote the catabolism of fat. The reason may be that dietary LPL supplementation affects the expression of lipoprotein transport (such as lipoprotein lipase and HL) genes for lipid metabolism [59].

## 5. Conclusions

The supplementation of the beef cattle diet with LPLs improved growth performance, feed efficiency, and apparent digestibility in a dose-dependent manner. Additionally, dietary LPL inclusion decreased fecal N excretion and urinary N excretion, elevated antioxidant levels in the blood, and regulated plasma metabolites in beef cattle. The results indicate that LPLs have the potential to be used as feed additives in beef cattle production.

## Figures and Tables

**Table 1:** Ingredients and nutrient compositions of the dietary treatments.

Item	Diet ^1^			
	CON	LLPL	MLPL	HLPL
Ingredient composition, (g/kg DM)				
Corn grain	460	460	460	460
soybean meal	50	50	50	50
Peanut hull	100	100	100	100
Soybean hull	50	50	50	50
Chinese wild ryegrass	100	100	100	100
Distiller-dried grains with solubles	120	120	120	120
Calcium bicarbonate	5	5	5	5
Corn germ meal	50	50	50	50
Rumen-protected fat	25	25	25	25
Molasses	5	5	5	5
Salt	7	7	7	7
Limestone	11	10.9	10.8	10.6
Magnesium oxide	3	3	3	3
Sodium bicarbonate	10	10	10	10
Mineral–vitamin premix ^2^	4	4	4	4
Lysophospholipids	0	0.12	0.24	0.48
Chemical composition				
OM, (g/kg DM)	921	923	922	924
CP, (g/kg DM)	115	116	116	117
Dry matter (DM), (g/kg)	886	891	885	892
Ether extract, (g/kg DM)	66	66	68	69
NDF, (g/kg DM)	261	261	263	260
ADF, (g/kg DM)	158	157	159	158
Ca, (g/kg DM)	7.7	7.8	7.8	7.9
*p*, (g/kg DM)	3.9	3.8	3.9	3.8
ME, (MJ/kg DM) ^3^	11.8	11.8	11.8	11.8

^1^ CON = control; LLPL = 0.012% lysophospholipids; MLPL = 0.024% lysophospholipids; HLPL = 0.048% lysophospholipids. ^2^ The mineral–vitamin premix provided the following per kilogram of the diet: VA, 6000 IU; VD, 600 IU; VE, 50 IU; Fe, 10 mg; Cu, 15.0 mg; Mn, 27 mg; Zn, 65 mg; I, 0.50 mg; and Co, 0.20 mg. ^3^ Estimated according to NRC (2000) [15].

**Table 2:** Effect of dietary lysophospholipid supplementation on the feed intake, growth performance, and digestibility of beef cattle.

Item ^1^	LPL Addition ^2^						*p*-Value	
	CON	LLPL	MLPL	HLPL	SEM ^3^	Treatment	Linear	Quadratic
ADG, (kg/d)	1.26 ^b^	1.32 ^b^	1. 55 ^a^	1.56 ^a^	0.086	<0.01	<0.01	0.66
FCR	8.52 ^a^	8.03 ^a^	6.70 ^b^	6.67 ^b^	0.030	<0.01	<0.01	0.52
DMI, (kg/d)	10.7	10.6	10.5	10.6	0.35	0.32	0.79	0.88
ME, (MJ/d)	126	126	125	125	4.1	0.30	0.79	0.88
Digestibility (g/kg DM)								
DM	615 ^c^	662 ^ab^	649 ^bc^	692 ^a^	13.0	<0.01	<0.01	0.88
EE	646 ^b^	716 ^a^	720 ^a^	753 ^a^	11.6	<0.01	<0.01	0.12
CP	564 ^b^	603 ^a^	601 ^a^	631 ^a^	14.2	<0.01	<0.01	0.74
NDF	369	355	361	347	9.1	0.40	0.15	0.99
ADF	238	232	242	250	7.3	0.38	0.19	0.36

^a,b,c^ Means within a row with different superscripts differ (*p* < 0.05). ^1^ ADG, average daily gain; DMI, dry matter intake; FCR, feed conversion ratio; DM, dry matter; EE, ether extract; CP, crude protein; NDF, neutral detergent fiber; ADF, acid detergent fiber. ^2^ CON = control; LLPL = 0.012% lysophospholipids; MLPL = 0.024% lysophospholipids; HLPL = 0.048% lysophospholipids. ^3^ SEM, standard error of means.

**Table 3:** Effect of dietary lysophospholipid supplementation on nitrogen utilization in beef cattle.

Item ^1^	LPL Addition ^2^						*p*-Value	
	CON	LLPL	MLPL	HLPL	SEM ^3^	Treatment	Linear	Quadratic
N intake, (g/d)	200	198	198	199	6.5	0.10	0.96	0.84
Fecal N excretion (g/d)	86.5 ^a^	77.7 ^b^	78.3 ^b^	75.9 ^b^	3.93	0.05	0.02	0.27
Fecal N excretion (g/kg of N intake)	431	390	394	380	5.8	<0.01	<0.01	0.02
Urinary N excretion (g/d)	64.5	56.3	54.5	55.1	4.26	0.10	0.04	0.18
Urinary N excretion (g/kg of N intake)	332	289	279	281	12.3	0.16	0.06	0.25
N retained (g/d)	49.0 ^b^	64.8 ^a^	65.6 ^a^	68.7 ^a^	6.37	0.04	0.01	0.24
N retained (g/kg of N intake)	236 ^b^	320 ^a^	325 ^a^	338 ^a^	13.6	<0.01	<0.01	0.07

^a,b^ Means within a row with different superscripts differ (*p* < 0.05). ^1^ N, nitrogen; ^2^ CON = control; LLPL = 0.012% lysophospholipids; MLPL = 0.024% lysophospholipids; HLPL = 0.048% lysophospholipids. ^3^ SEM, standard error of means.

**Table 4:** Effect of dietary lysophospholipid supplementation on the blood metabolic parameters of beef cattle.

Item ^1^	LPL Addition ^2^						*p*-Value	
	CON	LLPL	MLPL	HLPL	SEM ^3^	Treatment	Linear	Quadratic
TP, (g/L)	67.2	69.0	70.2	70.3	0.87	0.06	0.01	0.33
ALB, (g/L)	32.4 ^b^	33.8 ^a^	33.6 ^a^	31.7 ^b^	0.29	<0.01	0.06	<0.01
GLB, (g/L)	34.6	36.0	35.4	35.5	0.72	0.53	0.51	0.34
TG, (mmol/L)	0.21 ^a^	0.19 ^b^	0.19 ^b^	0.18 ^b^	0.007	0.02	<0.01	0.18
CHOL, (mmol/L)	4.60 ^a^	4.33 ^b^	4.19 ^b^	4.12 ^b^	0.115	0.04	<0.01	0.38
LDL-C, (mmol/L)	0.64 ^a^	0.61 ^b^	0.54 ^c^	0.52 ^c^	0.009	<0.01	<0.01	0.65
HDL-C, (mmol/L)	2.62 ^c^	2.71 ^b^	2.67 ^b^	2.78 ^a^	0.015	<0.01	<0.01	0.68
AST, (U/L)	63.5	66.6	64.4	66.9	2.15	0.61	0.42	0.88
ALP, (U/L)	129	140	142	140	5.7	0.38	0.20	0.25
ALT, (U/L)	16.5	19.0	17.6	17.7	1.06	0.25	0.66	0.27
Creatinine, (μmol/L)	88.0	85.7	90.7	91.0	3.26	0.60	0.34	0.69
GLU, (mmol/L)	4.78	4.78	4.79	4.77	0.117	0.10	0.97	0.96
UA, (μmol/L)	9.99	11.2	10.8	11.5	0.56	0.36	0.10	0.67
BUN, (mmol/L)	2.94	3.45	3.21	3.08	0.205	0.26	0.85	0.13

^a,b,c^ Means within a row with different superscripts differ (*p* < 0.05). ^1^ TP, total protein; ALB, albumin; GLB, globulin; TG, triglyceride; CHOL, cholesterol; LDL-C, low-density lipoprotein cholesterol; HDL-C, high-density lipoprotein cholesterol; AST, aspartate aminotransferase; ALP, alkaline phosphatase; ALT, alanine aminotransferase; GLU, glucose; UA, uric acid; BUN, urea nitrogen. ^2^ CON = control; LLPL = 0.012% lysophospholipids; MLPL = 0.024% lysophospholipids; HLPL = 0.048% lysophospholipids. ^3^ SEM, standard error of means.

**Table 5:** Effect of dietary lysophospholipid supplementation on the antioxidant function and enzyme activities of beef cattle.

Item ^1^	LPL Addition ^2^						*p*-Value	
	CON	LLPL	MLPL	HLPL	SEM ^3^	Treatment	Linear	Quadratic
T-AOC, (U/mL)	5.60	5.93	5.79	5.98	0.135	0.16	0.11	0.60
MDA, (nmol/mL)	3.33 ^a^	2.50 ^b^	2.52 ^b^	2.34 b	0.097	<0.01	<0.01	<0.01
T-SOD, (U/mL)	61.8	60.7	61.1	63.1	0.65	0.16	0.51	0.03
GSH-Px, (U/mL)	108	115	113	118	2.9	0.10	0.04	0.86
TL, (U/mL)	4.07 ^c^	5.76 ^b^	5.70 ^b^	6.87 ^a^	0.148	<0.01	<0.01	0.09
lipoprotein lipase, (U/mL)	1.52 ^b^	2.40 ^a^	2.45 ^a^	2.48 ^a^	0.058	<0.01	<0.01	<0.01
HL, (U/mL)	2.55 ^c^	3.37 ^b^	3.22 ^b^	4.41 ^a^	0.122	<0.01	<0.01	0.12

^a,b,c^ Means within a row with different superscripts differ (*p* < 0.05). ^1^ T-AOC, total antioxidant capacity; MDA, malondialdehyde; T-SOD, total superoxide dismutase; GSH-Px, glutathione peroxidase; TL, total lipase; HL, hepatic lipase. ^2^ CON = control; LLPL = 0.012% lysophospholipids; MLPL = 0.024% lysophospholipids; HLPL = 0.048% lysophospholipids. ^3^ SEM, standard error of means.

## Data Availability

The data are contained within the article.
